# The Black Box as a Control for Payoff-Based Learning in Economic Games

**DOI:** 10.3390/g13060076

**Published:** 2022-11-16

**Authors:** Maxwell N. Burton-Chellew, Stuart A. West

**Affiliations:** 1 Department of Economics, University of Lausanne, CH-1015 Lausanne, Switzerland; 2 Department of Biology, University of Oxford, Oxford OX1 3RB, UK

**Keywords:** altruism, asocial control, behavioural economics, conditional cooperation, confusion, directional learning, reinforcement learning, social preferences

## Abstract

The black box method was developed as an “asocial control” to allow for payoff-based learning while eliminating social responses in repeated public goods games. Players are told they must decide how many virtual coins they want to input into a virtual black box that will provide uncertain returns. However, in truth, they are playing with each other in a repeated social game. By “black boxing” the game’s social aspects and payoff structure, the method creates a population of self-interested but ignorant or confused individuals that must learn the game’s payoffs. This low-information environment, stripped of social concerns, provides an alternative, empirically derived null hypothesis for testing social behaviours, as opposed to the theoretical predictions of rational self-interested agents (*Homo economicus*). However, a potential problem is that participants can unwittingly affect the learning of other participants. Here, we test a solution to this problem in a range of public goods games by making participants interact, unknowingly, with simulated players (“computerised black box”). We find no significant differences in rates of learning between the original and the computerised black box, therefore either method can be used to investigate learning in games. These results, along with the fact that simulated agents can be programmed to behave in different ways, mean that the computerised black box has great potential for complementing studies of how individuals and groups learn under different environments in social dilemmas.

## 1 Introduction

Understanding human behaviour in social dilemmas is of crucial importance to solving many global issues [1–7]. Experiments using economic games provide a useful tool for investigating social behaviours [8]. By making participants pay for their decisions, experimenters hope to measure social preferences on the assumption that participants pay for preferred outcomes [9]. By using games with repeated decisions (repeated games), experimenters hope to measure how individuals respond to the behaviours of others (social responses) [10–18]. However, in repeated games, social responses can be confounded by individuals responding to their payoffs and learning how to play the game (payoff-based learning) [19–23]. This problem is particularly acute if many participants start the experiment without fully understanding the game’s payoffs [24–28]. Consequently, experimental control treatments are required to control for potentially confounding factors such as payoff-based learning.

One solution to this problem of confounding is to make individuals face the same decision but in a low-information environment stripped of all social concerns (“asocial controls”) [19,25,27,29–33]. For example, Burton-Chellew et al. introduced the black box method as an “asocial control” to decouple social responses from payoff-based learning in repeated public-goods games [19–21,34]. Specifically, individuals interacted with a virtual black box, with which they could make voluntary inputs of “virtual coins” to obtain uncertain returns over several rounds^1^. However, in reality, the experiment actually involved groups of real participants playing a typical public goods game, with all the usual payoffs and social connections, just unknowingly^2^. By repeating the black box game for multiple rounds, one could measure how inputs evolved in populations of ignorant individuals with no social concerns [19–21]. The black box thus aimed to capture the psychology of self-interested but ignorant/confused individuals that use trial and error learning to improve their earnings. In this way, it was consistent with a rich history of prior studies that investigated how individuals learn in low-information environments and how reinforcement learning can affect cooperation [35–42].

The black box as an asocial control provided an alternative null hypothesis, empirically derived from behavioural observations, to the usual theoretical null hypotheses of a population of perfectly rational and selfish agents (*Homo economicus*). This “baseline” measure could then be compared to behaviour in versions of the normal, “revealed” public goods game to test if the addition of social information affected aggregate behaviour. Burton-Chellew and West’s original results showed that aggregate contributions in the black box treatment were largely indistinguishable from those in the standard “revealed” public goods game, where individuals can observe their groupmates’ decisions, consistent with models of payoff-based learning [19]. In both cases, despite the income maximising decision in the one-shot version of the game being to contribute 0%, initial levels of contributions averaged around 40–50%, before gradually declining to approximately 15% by round 16, the final round. In both cases, most individuals contributed 0% in the final round, but approximately four percent of individuals still contributed fully. While these similarities did not confirm that individuals were using payoff-based learning, they did mean that one could not reject the null hypothesis of self-interest unless one assumed the participants perfectly understood the revealed game and that the similar levels of cooperation were mere coincidence.

Although there were large similarities between the black box results and the typical results, it is important to keep in mind that the black box was providing a simplified model of behaviour based on the extreme assumption that all players are ignorant/confused and respond only to their own payoffs [19]. However, the black box also allows for examinations of how individuals learn and for estimating parameters within explicit hypothesised learning rules [20,21]. For example, subsequent collaborations with H. Nax and H. Peyton Young analysed individual-level data to estimate how much individuals value the earnings of their groupmates [20] and how individuals use payoff-based learning in the non-social and two social settings [21]. Not surprisingly, some differences in behaviour were found across the three treatments (the black box is, after all, like *Homo economicus*, a rather extreme hypothesis/model). When individuals could observe their groupmates’ decisions, they showed some conditional responses, but only if they could not also observe their groupmates’ payoffs (which is technically redundant information if individuals fully understand the game). However, payoff-based learning was significant in all three treatments [20], including even the manner of such learning [21], suggesting participants were motivated to try and increase their own income in all game forms. Together, these results suggested that conditional cooperation was more a function of social learning rather than a social preference for equal outcomes, although learning and social preferences may also interact [43].

The black box can also be easily modified and adapted to test different hypotheses. For example, Burton-Chellew and West [32] also subsequently used the black box to show that payoff-based learning is impeded when either group size (N) or the marginal per capita return from contributing is large (MPCR). By testing behaviour in three different black boxes that varied in either group size (N = 3 or 12) or the marginal per capita return from contributing (MPCR = 0.4 or 0.8), they showed that a large group size and/or a high MPCR and thus reduces the correlation between personal contributions and personal payoffs, thereby impeding payoff-based learning and potentially explaining why the rate of decline in cooperation varies across studies. They confirmed this hypothesis with a comparative analysis that compared the rates of decline in 237 published public goods games. They found that rates of decline in contributions were slower when either group size or MPCR was large, and more specifically, when the estimated correlation between personal contributions and personal payoffs was weaker, a principle proved in their black box experiment [32].

However, one potential issue with the black box is that because participants are interacting, albeit unknowingly, the learning of one participant changes the learning environment for other participants. While this is also true for revealed social games, it may complicate efforts to discern individual learning from collective learning [44]. Another possible issue is that individuals do not know they can provide benefits to other participants, which may raise ethical concerns for some reviewers (however we do not think this omission of externalities constitutes deception).

**Figure 1:**
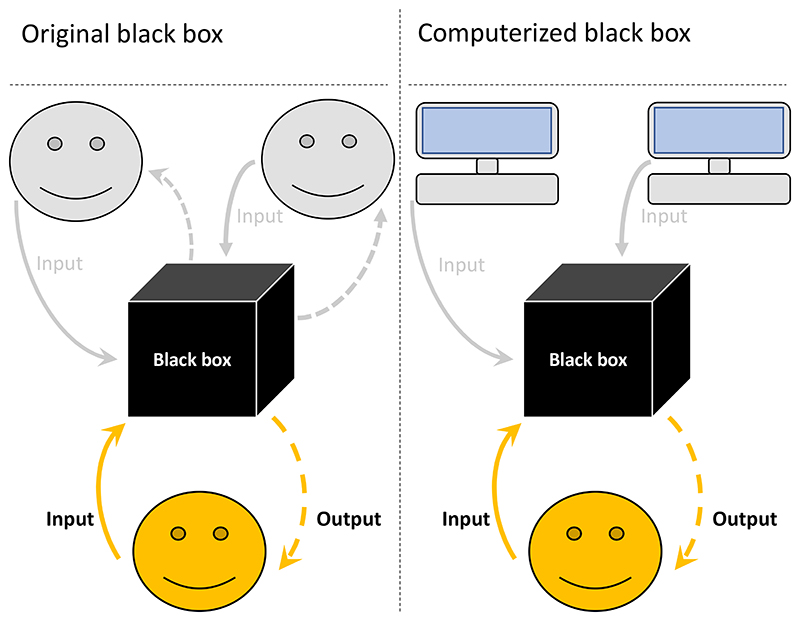
Black box methodologies. In the original black box, participants are connected online and interact in the usual experimental manner for economic games. However, by “black-boxing” the social aspect of the game or the game’s rules and payoffs, one can investigate how participants learn under certain conditions. If one is concerned about individuals affecting either the learning or the payoffs of other participants, one can replace the focal player’s interaction partners with programmed computerised/virtual players (computerised black box). This also allows for more control over the learning environment, as partners can be programmed to be more/less cooperative, etc.

Here, we present a modified black box method that solves these two potential issues. Our solution is to make individuals still interact with a black box but change the set-up so that individuals are grouped not with each other but with computerised players (computerised black box) (Figure 1). Otherwise, the set-up remains the same for the participants. There are several advantages to this approach: (1) individuals do not affect other participants, and thus can be treated as independent data points, providing more statistical power for given costs; (2) the learning environment can be maintained constant; (3) individuals are not affecting each other’s payoffs, thereby removing any potential ethical concerns; (4) computerized players receive no earnings making the study of behaviour in large groups more affordable; and (5) computerised players can be programmed to play in different, interesting ways, allowing one to test various hypotheses that would otherwise be unfeasible without using deception.

We replicate the experimental design from Burton-Chellew and West, 2021, which used three different black boxes that varied in either group size or the cost of contributing to create one “easy” learning condition with a small group size and low MPCR (N = 3 and MPCR = 0.4) and two “difficult” learning conditions with either a large MPCR (N = 3 and MPCR = 0.8) or a large group size (N = 12 and MPCR = 0.4) [32]. Individuals could input 0–20 virtual coins in each round. However, instead of connecting human participants together, here we use computerised groupmates (programmed to input a random integer drawn from a uniform distribution of 0–20 coins). The payoff formula remained identical for all rounds and was the same for the human and computerised black boxes. This allowed us to compare rates of learning in the two methods, depending on both group size and MPCR. If behaviour with computerised black boxes qualitatively replicates behaviour with human black boxes, then the computerised black box method can be used as a complementary method to test hypotheses without any concerns about participants affecting each other’s behaviour and/or earnings.

We also address a related research question on payoff-based learning. As mentioned above, Burton-Chellew and West, 2021, previously showed that payoff-based learning is impeded when groups are large or MPCR is high [32]. In such conditions, participants still contributed around 50% at the end of 16 rounds, despite the Nash equilibrium being 0%, indicative of zero learning. Nevertheless, it may be that individuals just need more time to learn in these challenging conditions and will eventually learn not to contribute. To test this, we repeated the black boxes with the difficult learning conditions, but under two conditions, a short game and a long game (16 versus 40 rounds).

In all cases, we measured learning in two ways; (1) how quickly incentivised contributions converged towards the Nash equilibrium of 0 contributions, and (2) by asking participants at the end of the experiment to report their belief about what was the best number to contribute (“input”) into the black box (this was unincentivised).

## 2 Results

### 2.1 Learning with Hidden Humans or Hidden Computers

**Figure 2:**
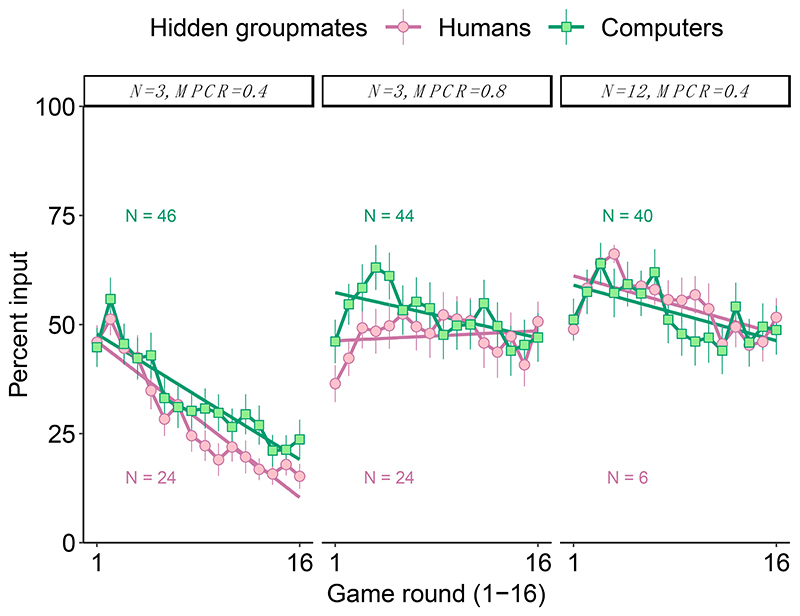
Learning in a black box, either with hidden human or computerised groupmates. We varied both the group size (N) and the benefit of contributing (MPCR) across three black boxes. Participants played all three black boxes, in counter-balanced order, but here we only show naïve behaviour (their first black box). Data show the mean contribution per round, with 95% confidence intervals based on the group means (in games with computers, the independent group is just one individual). The rate of learning was broadly similar regardless of playing with humans or computers. The linear regressions do not account for random effects/repeated measures and are therefore for illustration purposes only. The figures are annotated with the sample sizes of independent replicates (groups of humans or individuals grouped with computers).

We found that there was no significant difference in contributions (“inputs”) depending upon whether individuals were grouped with humans or computers. The rate of decline in contributions across all 16 rounds of the short games did not significantly differ between the original black box with humans and the computerised black box in any of the three black boxes (Figure 2; Table 1). Specifically, the game round x groupmates interaction was non-significant in all three black boxes (generalised linear mixed models controlling for autocorrelation among groups/individuals: when N = 3 and MPCR = 0.4, Z = 0.2, *p* = 0.821; when N = 3 and MPCR = 0.8, Z = −1.2, *p* = 0.222; and when N = 12 and MPCR = 0.4, Z = −0.3, *p* = 0.760, Table 1).

As an additional check, we also compared final round contributions (“inputs”), which could be argued to be the best measure of learning. Again, we found no significant differences between playing with humans or with computers in any of the three black boxes (Table 2). Specifically, when learning was easy (N = 3 and MPCR = 0.4), mean ± SE final round inputs (0–20 virtual coins) were 3.0 ± 0.57 coins with humans and 4.7 ± 0.90 coins with computers (Wilcoxon rank-sum test: W = 537, *p* = 0.797). When learning was difficult, mean final round inputs were typically around 50% (10 virtual coins) with both humans and computerised groupmates (N = 3 and MPCR = 0.8, with humans = 10.1 ± 0.91 coins, with computers = 9.4 ± 1.12 coins, W = 573.5, *p* = 0.563; N = 12 and MPCR = 0.4, with humans = 10.3 ± 0.89 coins, with computers = 9.7 ± 1.12 coins, W = 128, *p* = 0.806).

**Figure 3:**
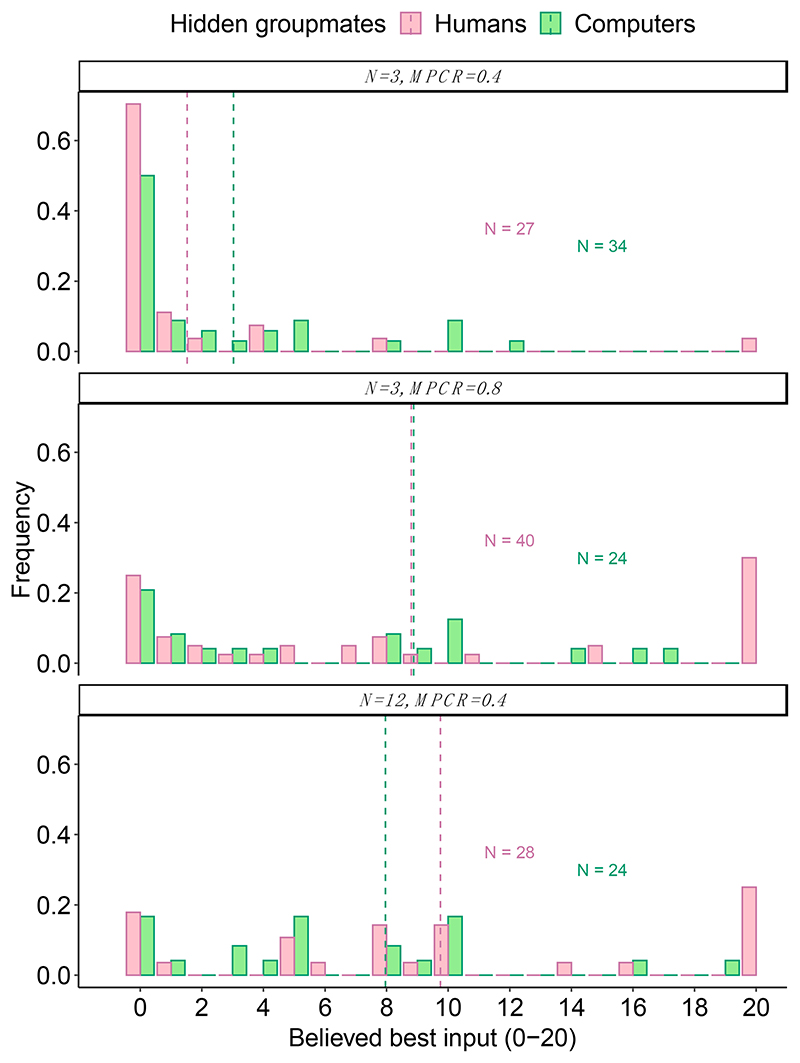
Groupmates and beliefs. Histograms show the frequency of each stated belief about what was the best number to input into the black box. Dashed vertical lines show the mean response. All responses are from naïve participants after finishing their first black box. The figures are annotated with the number of individuals.

We also asked the participants at the end of the 16 rounds if they thought there was a best number to input and if so, what it was (methods). Again, there were no significant differences depending on whether playing with humans or with computers (Figure 3; Table 3). Specifically, the mean ± SE stated beliefs (0–20 coins) for when N = 3 and MPCR = 0.4 were 1.5 ± 0.79 coins with humans and 3.0 ± 0.80 coins with computers (Wilcoxon rank-sum test, W = 348, *p* = 0.072); for when N = 3 and MPCR = 0.8, they were 8.8 ± 1.32 coins with humans and 8.9 ± 1.59 coins with computers (W = 469.5, *p* = 0.888); and for when N = 12 and MPCR = 0.4, were 9.8 ±1.38 coins with humans and 8.0 ± 1.37 coins with computers (W = 387, *p* = 0.349).

Overall, we found no significant differences between either inputs or beliefs, depending on if individuals were grouped with humans or computers. Our experiments with computerised groupmates replicated the results from the prior study with human group-mates [32]. Rates of learning were qualitatively similar regardless of groupmates being humans or computers in all three black box settings (Figure 2). These results mean that the original black box with humans can be used without having to worry too much about collective learning, or alternatively that the new, computerized, black box method can be used in certain contexts to obtain qualitatively similar results. However, we caution that for the “easy” black box (N = 3 and MPCR = 0.4), the final round inputs and the post-game beliefs about the value of the best input were lower, but not significantly, in the human black box. Looking at Figure 2, it may be that the learning rates for the “easy” black box (N = 3 and MPCR = 0.4) would have diverged if the experiment had continued for longer than 16 rounds, but we find no statistical support for this prediction within our data.

### 2.2 Learning in Longer Games

**Figure 4:**
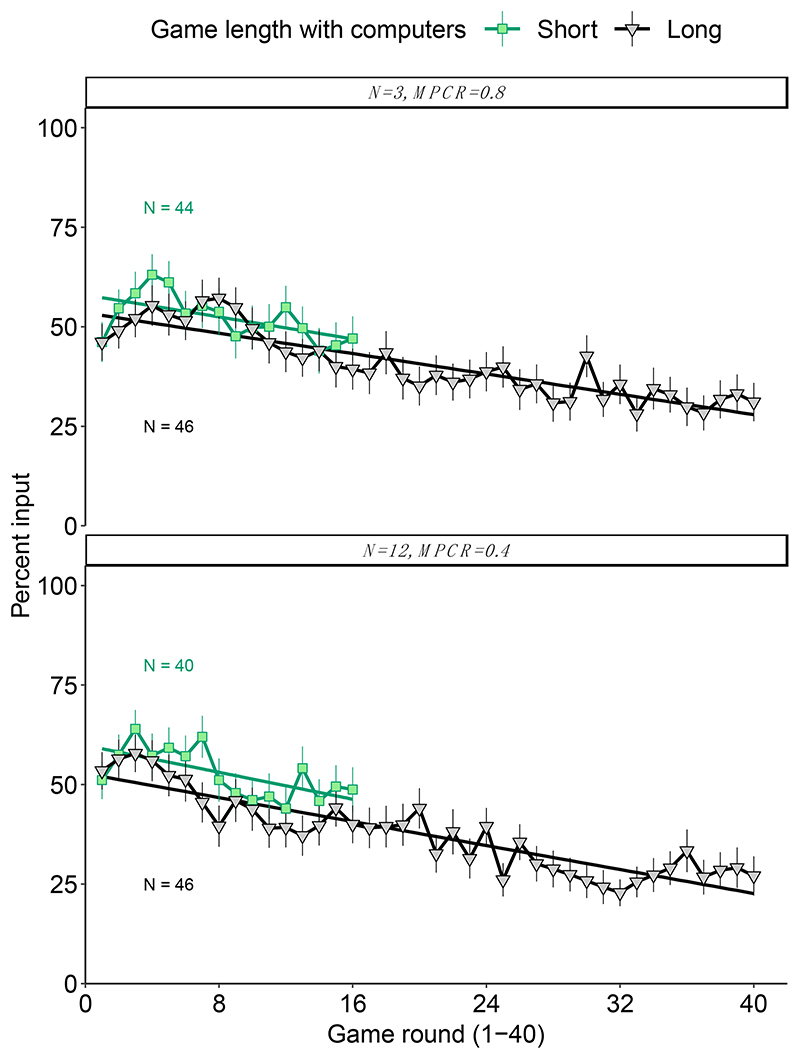
Learning and game length. The green data are the same as in Figure 2. Data show mean contributions with 95% confidence intervals, depending on game length, for two different black box parameter settings. The rate of learning was broadly similar in both black boxes regardless of game length, but because learning is slow in these parameter settings (large groups or high MPCR), the learning is only evident in long games. The linear regressions do not account for random effects/repeated measures and are therefore for illustration purposes only. The figures are annotated with the number of independent replicates (individuals grouped with computers).

We found clear evidence of payoff-based learning in the long-run games (Figure 4). Overall, the estimated rate of decline was significantly negative in both black boxes (Table 4, generalised linear mixed model controlling for individual: when N = 3 and MPCR = 0.8, Z = −2.8, *p* = 0.005; when N = 12 and MPCR = 0.4, Z = −3.3, *p* < 0.001, depending on black box). However, for both black boxes, the rate of decline was not significantly different between the short and long games, suggesting that the rate of learning is relatively constant within these time frames despite being undetectable in the short games (Table 4, round x game length interaction: N = 3 and MPCR = 0.8, Z = 1.0, *p* = 0.308; N = 12 and MPCR = 0.4, Z = 0.5, *p* = 0.643).

Again, we compared the mean final contributions (“inputs”). These were significantly smaller, and thus closer to the income-maximising input of 0 coins, after the long game than after the short game in both black boxes (Table 2). Specifically, for the black box, where N = 3 and MPCR = 0.8, mean ± SE final inputs were 9.4 ± 1.12 coins in the short game and 6.2 ± 0.97 coins in the long game (Wilcoxon rank-sum test, W = 1287.5, *p* = 0.025). For the black box where N = 12 and MPCR = 0.4, final inputs were 9.7 ±1.12 coins in the short game and 5.4 ± 0.98 coins in the long game (W = 1238.5, *p* = 0.006).

**Figure 5:**
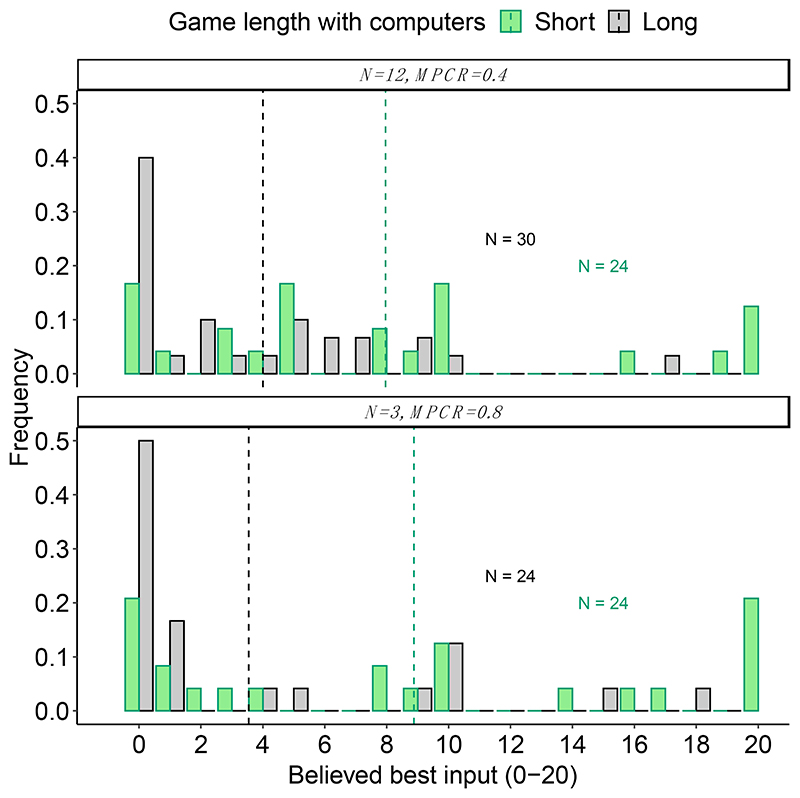
Game length and beliefs. Histograms show the frequency of each stated belief about what was the best number to input into the black box. Dashed vertical lines show the mean response. Beliefs were more accurate after a longer game. All responses are from naïve participants after finishing their first black box. The figures are annotated with the number of individuals.

Moreover, when asked about their beliefs about a possible best number, stated beliefs were significantly lower on average at the end of the long games compared to the short games (Figure 5, Table 3). Specifically, the mean ± SE stated beliefs (0–20 coins) for when N = 3 and MPCR = 0.8, were 8.9 ± 1.59 coins in the short game and 3.5 ± 1.10 coins in the long game (Wilcoxon rank-sum test, W = 410.5, *p* = 0.010); for when N = 12 and MPCR = 0.4, were 8.0 ± 1.37 coins in the short game and 4.0 *±* 0.93 coins in the long game (W = 496.5, *p* = 0.016).

## 3 Discussion

### 3.1 Payoff-Based Learning in Public-Goods Games

Our results show clear evidence that participants are capable of payoff-based learning in public goods games (Figures 2 and 4). Such learning does not require being “collective”, because we found strong evidence of learning even when we grouped individuals with computerised players that just played randomly and did not learn (Figure 2). Overall, we found clear evidence for payoff-based learning towards the income-maximising decision in both our short and long form games, suggesting that even when the learning environment is difficult, e.g., when group sizes are large (N = 12), or when the costs of “wrong” decisions are relatively small, e.g., when MPCR = 0.8, individuals can still learn to improve their income (Figure 4).

It has long been appreciated that participants will likely need time to learn in economic experiments [18,45–49]. While the role of learning has been clear in non-social dilemma studies as an explanation for initial non-income-maximising behaviour, it has often been disputed in social games [50]. Instead, many researchers have assumed that their participants understood the game from the beginning and were instead responding to other participants rather than their payoffs [16,51]. However, it is interesting to note that the standard procedure in repeated games is to show participants their payoffs after each round, which begs the question of why this was assumed necessary.

### 3.2 The Value of Control Treatments in Economic Experiments

One reason for this difference in approach between non-social and social experiments could be because deviations from income-maximising behaviour can always be rationalised in social games as behaviour motivated by the social consequences [9,52]. However, an approach of simply “measuring” social behaviours and preferences is problematic because it does not control for other behavioural processes, such as payoff-based learning.

Instead, when attempting to measure social behaviours, especially in artificial settings, one needs adequate behavioural controls to test whether the social factors are motivating behaviour. For instance, one can enhance how information is presented or framed to see if this affects social behaviours. This can be a particularly powerful approach when the information is technically redundant and thus provides no new information to participants yet still affects social behaviours [19,23].

Structurally, one can change the game’s payoffs so that failures to maximise income harm rather than benefit social partners. Such a reversal of social consequences should largely eliminate failures to maximise income among truly prosocial participants, a hypothesis falsified by studies that converted public-goods games into “public-delight” games [19,26,53,54]. In public-delight games, the return from contributing is set to be greater than 1, meaning that both selfish and prosocial individuals should contribute fully [19,26,53,54]. However, results show that many individuals still initially fail to maximise income before learning to moderately increase (rather than decrease) their contributions as the game is repeated, a result not easily explained by any rational social preferences [19,26,53,54]. Two reasons the increase may only be moderate when the MPCR > 1 are that in the public-delight game (1) the personal benefit of contributing is easily dwarfed by the benefits obtained from groupmates’ contributions, meaning that individuals have relatively less influence over their own payoff, impeding payoff-based learning [32]; and (2) contributions always produce profits, meaning individuals never suffer losses, which perhaps are more salient mistakes than failures to maximise profits.

Alternatively, one can remove social factors to create “asocial controls”, either by presenting the game differently, as is done by the black box, or structurally, by using revealed games played with computerised partners, which eliminate social concerns. There is a long history of using games with computerised partners, and such experiments have been useful to show that apparently social behaviours are not unique to games with human partners [25,27,29,30,33]. However, the interpretation of games with computers can be disputed, because although they clearly show that individuals often fail to maximise income even when there are no social consequences, they cannot rule out that individuals are psychologically motivated to help even computers.

The black box method provides a complementary form of asocial control to games with computers because, instead of removing social interactions, it hides them in a low information environment [19,31]. Consequently, the game is so devoid of social factors and framing that it is implausible to argue participants are behaving according to social psychology. Instead, the black box provides a clean measure of how a population of self-interested participants will collectively learn with experience, which can serve as a useful baseline measure of what behaviour to expect: if the addition of social information does not amplify social responses, then it may be more parsimonious to assume payoff-based learning is responsible. Specific learning hypotheses can then be tested with individual level data [20,21]. If one is concerned about participants in the original version of the black box affecting the learning and earnings of other participants, one can use the computerised black box to obtain similar results. One can even test various hypotheses by programming how the computerised partners will behave. One could then test if and how such learning spills over into other games or games with real humans, perhaps black box learning could be used to improve cooperation in games where cooperation is favoured [55].

## 4 Materials and Methods

### 4.1 Participants and Location

We analysed previously published data from Burton-Chellew and West, 2021 [32], and compared these data with those from a new experiment with computerised black boxes. The new experiment was conducted in February 2020 using 222 participants across 14 sessions over 3 days at the Centre for Experimental Social Sciences, Oxford (CESS). CESS recruited the participants from their entire database with the sole restriction that they could not have participated in a prior study by Burton-Chellew and West, 2021 [32]. CESS deployed assistants to manage participant reception, consent, and payments. MNBC conducted the experiment in a laboratory with 25 computer stations. For each session, we made 25 spaces available, and attendance varied from 7 to 25. The experiment was coded and conducted in z-TREE and participants were recruited using the ORSEE software [56,57].

The total sample was 222 participants. Their age ranged from 18 to 81 years old. The mean ± SD was 31.8 years *±* 15.9 (N = 192, this does not include seven participants that declined to answer nor all 23 participants from the first session). According to the self-reported genders, we had 139 females, 81 males, one other and one declined to answer.

### 4.2 Experiment Design

A copy of the instructions is available in Appendix A and online at the Open Science Framework, along with the consent form, data and analysis script. Our design replicated the three “short” treatments from Burton-Chellew, 2021, which varied both the group size (N) and the return from contributing (Marginal Per Capita Return, MPCR). We also added two “long” treatments, played for 40 rounds instead of 16 rounds. However, instead of connecting real individuals with each other, we connected each participant with N-1 computerised virtual players that simulated random decisions drawn from a uniform decision. This way, the payoff was entirely consistent with a public goods game, but players had no knowledge of the game, and there were no ethical concerns about possible deception or a lack of informed consent.

Participants either played all three short treatments or one long treatment. When playing all three short treatments, we counterbalanced the order across sessions (although not perfectly, as the six permutations required a multiple of six sessions and we had eight). However, here we only analyse the “naïve” data from participants playing their first game. The reason we made participants play all three short black boxes was to standardise payments across sessions (a higher MPCR leads to increased mean payoffs).

The five treatments and naïve sample sizes were: black box with N = 3 and MPCR = 0.4 first = 46 (over three sessions, 23, 11, 12); black box with N = 3 and MPCR = 0.8 first = 44 (over three sessions, 22, 12, 10); black box with N = 12 and MPCR = 0.4 first = 40 (over two sessions, 18 and 22); black box with N = 3 and MPCR = 0.8 long version = 46 (over three sessions, 22, 7, 17); black box with N = 12 and MPCR = 0.4 long version = 46 (over three sessions, 25, 9, 12).

The exchange rate was 0.65 pence to 1 virtual coin, except in the first session, where it was 0.75. The total endowment was 960 coins (GBP 6.24) in the short treatment sessions and 800 coins (GBP 5.20) in the long treatments. Payments were rounded up to the nearest 10 pence. Earnings ranged from GBP 8.20 to GBP 16.90 (mean = GBP 12.38), to which a GBP 5 show up fee was added.

After each black box, we asked participants two questions: (1) “Do you think there was a best number to put in this black box? Please enter 1 for ‘Yes’ and 0 for ‘No’.”; and then (2) “If yes, what do you think was the best number (0–20)? If no, then please enter 99.” Then after the experiment, we asked the participants to complete a brief survey on their self-reported gender, age, and personality traits, before asking them, “In a few words, please tell us what, if anything, you think the experiment was about?”. We did this to check that they did not perceive the experiment as a social dilemma. As responses to this same question have already been analysed by Burton-Chellew and West, 2021, who found that only 2% (N = 5/216) of participants (N = 5/216) “mentioned anything that could be construed as social”, we do not analyse the responses here [32].

### 4.3 Analyses

All tests are two-tailed and we conducted all analyses in RStudio [58]. All our data and analysis files are freely available online at the Open Science Framework, https://osf.io/9uxdv/ [59].

## Supplementary Material

Appendix

## Figures and Tables

**Table 1:** Contributions over time. Analysis of how contributions (“inputs”) change during the game for each black box depending on if groupmates were humans or computers. Generalised linear mixed model with a binomial logit link, and random intercepts for both groups and individuals and random slopes for individuals.

	N = 3, MPCR = 0.4		N = 3, MPCR = 0.8		N = 12, MPCR = 0.4	
Fixed Effects	Z	p	Z	p	Z	p
Intercept (humans)	0.7	0.476	−1.4	0.159	3.6	<0.001
Round	−8.0	<0.001	2.0	0.046	−1.0	0.298
Groupmates (computers)	0.3	0.795	2.2	0.030	−0.1	0.896
Round x Groupmates	0.2	0.821	−1.2	0.222	−0.3	0.760
N. obs.	1888		1856		1792	
N. individuals	118		116		112	
N. groups	70		68		46	
Random effects	Variance	St. dev.	Variance	St. dev.	Variance	St. dev.
Individual intercept	1.12	1.059	1.36	1.168	3.00	1.733
0.3	0.167	0.07	0.255	0.02	0.170
Group intercept	0.60	0.777	1.08	1.038	0.00	0.00

**Table 2:** Final contributions. Comparison of mean final round contributions (“inputs”) of virtual coins into the black box (0–20 coins). Comparisons made with Wilcoxon rank-sum test.

Black Box	Input: Humans	Input: Computers (Short)	Input: Computers (Long)	W ^1^	P ^1^	W ^2^	P ^2^
N = 3, MPCR = 0.4	3.0 ± 0.57	4.7 ± 0.90	/	573	0.797	/	/
N = 3, MPCR = 0.8	10.1 ± 0.91	9.4 ± 1.12	6.2 ± 0.97	573.5	0.563	1287.5	0.025
N = 12, MPCR = 0.4	10.3 ± 0.89	9.7 ± 1.12	5.4 ± 0.98	128	0.806	1238.5	0.006

1 Comparing final inputs with human or computerised groupmates.

2 Comparing final inputs in short or long games.

**Table 3:** Beliefs about the best number. The mean ± SE value participants stated as the best number to input at the end of the game. Comparisons made with Wilcoxon rank-sum test.

Black Box	Humans (N) *	Computers— Short (N)	Computers— Long (N)	W ^1^	P ^1^	W ^2^	P ^2^
N = 3, MPCR = 0.4	1.5 ± 0.79 (27) *	3.0 ± 0.80 (34)	/	348	0.072	/	/
N = 3, MPCR = 0.8	0.88 ± 1.32 (40)	8.9 ± 1.59 (24)	3.5 ± 1.10 (24)	469.5	0.888	410.5	0.010
N = 12. MPCR = 0.4	9.8 ± 1.38 (28) *	8.0 ± 1.37 (24)	4.0 ± 0.93 (30)	387	0.349	496.5	0.016

* An error prevented data collection from some participants in the first seven sessions with human groupmates.

1 Comparing beliefs after play with human or computerised groupmates.

2 Comparing beliefs after short or long games.

**Table 4:** The effect of game length. Analysis of how inputs change during the game for each black box depending on game length (16 or 40 rounds). Generalised linear mixed model with a binomial logit link and random intercepts and slopes for individuals.

	N = 3, MPCR = 0.8		N = 12, MPCR = 0.4	
Fixed Effects	Z	p	Z	p
Intercept	1.1	0.257	1.7	0.080
Round	−2.8	0.005	−3.3	<0.001
Game length (short)	0.6	0.575	0.4	0.666
Round x Game length	1.0	0.308	0.5	0.643
N. obs.	2544		2480	
N. individuals	90		86	
N. groups	90		86	
Random effects	Variance	St. dev.	Variance	St. dev.
Individual intercept	3.00	1.731	5.67	2.381
Individual slope	0.01	0.116	0.01	0.122

## Data Availability

Data and analysis files are freely available online at the Open Science Framework, https://osf.io/9uxdv/ [59].

## Footnotes

1 Participants were told that the black box contained a mathematical function which would remain constant for the experiment, but which contained a random component each round, meaning that a given input would not guarantee the same output, but giving the impression that the black box was in some sense solvable.

2 Note that this is not deception as no false information is giving to participants. It is merely an omission of information about the externalities of the participant’s decisions. Crucially, participants are not going to leave the laboratory thinking that next time they play a game with humans that the humans are actually computers or actors (which is arguably the main reason for the no deception policy).
