# Supplementary material for: The Black Box as a Control for Payoff-Based Learning in Economic Games
Source: Games (Basel). Author manuscript; Available in PMC 2023 Jan 21. (PMC7614088; doi:10.3390/g13060076)
Supplement: Appendix [file EMS160016-supplement-Appendix.pdf]

Appendix A

A copy of the instructions for the sessions with three short black boxes. For the long games, the details were changed accordingly. Instructions were available on-screen and on a paper handout.

Welcome to the Experiment!

A copy of these instructions is also available on-screen.

We are going to give you some virtual coins. Each 'coin' is worth real money.

You are going to make a decision regarding the investment of these 'coins'.

This decision may increase or decrease the number of coins you have.

The more coins you have at the end of the experiment, the more money you will receive at the end.

At the end of the experiment the total amount of 'coins' you have earned will be converted to pennies at the following rate: 100 coins = 65 pennies, or = GBP 0.65.

In total, you will be given 960 coins GBP 6.24 with which to make decisions and your final total, which may be more or less than 960 coins, will depend on these decisions.

The Decision

You will face the same decision many times. Each time we will give you 20 virtual 'coins'. Then you must decide on how many of your 20 coins to input into a virtual 'black box'.

This 'black box' performs a mathematical function that converts the number of 'coins' inputted into a number of 'coins' to be outputted.

The mathematical function contains two components, one constant, deterministic, component which acts upon your input, and one 'chance' component.

You will play with this 'black box' for many rounds (more on this later), and the mathematical function will not change, but the chance component means that if you put the same amount of coins into the 'black box' over successive rounds, you will not necessarily get the same output each time.

The number outputted may be more or less than you put in, but it will never be a negative number, so the lowest outcome possible is to get 0 (zero) back.

If you chose to input 0 (zero) coins, you may still get some back from the black box.

All coins not inputted into the black box will be automatically 'banked' into your private account.

All coins outputted from the black box will also be 'banked' and go into your private account.

You will be paid all the coins from your private account at the end of the experiment.

So, in summary, your income from each decision will be the initial 20 coins, minus any you put into the 'black box', plus all the coins you get back from the 'black box'.

Playing the Same Box Many Times

You will play this game (make this decision) 16 times. Each time we will give you a new set of 20 coins to use.

Each Decision is Separate but the *'Black Box'* Remains the Same

This means you cannot play with money gained from previous decisions, and the maximum you can ever put into the 'black box' will be 20 coins.

And you will never run out of money to play with as we will give you a new set of coins for each decision.

Please see the attached figure overleaf for a summary of the experiment.

Playing with Different Boxes

After you have finished your 16 decisions, you will play again with a new 'black box'.

In total, you will play with 3 black boxes in the whole experiment.

All black boxes are the same in that they perform a mathematical function that converts the number of coins inputted into a number of coins to be outputted.

However Each Black Box Will Have a Different Mathematical Function

But the functions will always contain two components, one constant, deterministic, component, and one 'chance' component. You will play with this black box for many rounds, and the mathematical function will never change, but the chance component means that if you put the same amount of coins into the black box over successive rounds, you will not necessarily get the same output each time.

You Will Be Told When the Decisions are Finished and It Is Time to Play with a New Black Box

If you are unsure of the rules please hold up your hand and a demonstrator will help you.

Notes

1. Participants were told that the black box contained a mathematical function which would remain constant for the experiment, but which contained a random component each round, meaning that a given input would not guarantee the same output, but giving the impression that the black box was in some sense solvable.
2. Note that this is not deception as no false information is giving to participants. It is merely an omission of information about the externalities of the participant's decisions. Crucially, participants are not going to leave the laboratory thinking that next time they play a game with humans that the humans are actually computers or actors (which is arguably the main reason for the no deception policy).
